# Network analysis of monoamines involved in anxiety-like behavior in a rat model of osteoarthritis

**DOI:** 10.1007/s43440-023-00562-5

**Published:** 2024-01-05

**Authors:** Jakub Mlost, Magdalena Białoń, Marta Kędziora, Agnieszka Wąsik, Żaneta Michalec, Katarzyna Starowicz

**Affiliations:** Department of Neurochemistry, Maj Institute of Pharmacology, Polish Academy of Sciences, Ul. Smętna 12, 31-343 Kraków, Poland

**Keywords:** Chronic pain, Osteoarthritis, Depression, Anxiety, Monoamines, Nucleus accumbens

## Abstract

**Background:**

Chronic pain is a major health problem that affects a significant number of patients, resulting in personal suffering and substantial health care costs. One of the most commonly reported causal conditions is osteoarthritis (OA). In addition to sensory symptoms, chronic pain shares an inherent overlap with mood or anxiety disorders. The involvement of the frontal cortex, striatum and nucleus accumbens, in the affective processing of pain is still poorly understood.

**Methods:**

Male Wistar rats were divided into two groups: MIA (monoiodoacetate injected into the knee—model of OA) and sham (NaCl). Behavioral tests assessing pain, anxiety, and depressive behavior were performed at week 1, 3, 4, 6, 8, and 10. Neurochemical assays were conducted at weeks 3, 6, and 10 post-MIA injection, followed by the neurotransmitters and their metabolites correlation matrix and network analysis.

**Results:**

OA animals developed rapid pain phenotype, whereas anxiety-like behavior accompanied the development of a pain phenotype from 6 week post-MIA injection. We did not detect any depressive-like behavior. Instead, immobility time measured in the forced swimming test transiently decreased at 3 weeks post-MIA in the OA group. We detected changes in noradrenaline and serotonin levels in analyzed structures at distinct time points. Network analysis revealed noradrenaline and serotonin neurotransmission changes in the nucleus accumbens, confirming it to be the key structure affected by chronic pain.

**Conclusion:**

Animals with chronic pain exhibit symptoms of anxiety-like behavior and we identified underlying neurochemical changes using network analysis.

**Supplementary Information:**

The online version contains supplementary material available at 10.1007/s43440-023-00562-5.

## Introduction

Chronic pain remains an economic, sociological, and personal burden affecting up to 30% of the population [1]. The situation continues to worsen with the aging society and the increasing incidences of lifestyle disorders [2]. Notably, chronic pain, appearing as a result of a particular injury or a disease, is rather a separate medical condition [3]. The development of chronic pain can be caused by osteoarthritis (OA), a degenerative joint disease in which irreversible cartilage, synovial membrane, and subchondral bone degradation are observed [4]. Data suggest that OA patients are more vulnerable to pain, anxiety, and mood swings [5], significantly increasing the number of general practitioner visits and health care use, drug prescriptions, and postsurgical pain [6]. Studies have reported that over 60% of chronic pain patients suffer from psychiatric conditions [7] and 35% show symptoms of depression [8]. Moreover, chronic pain and depression are the most frequently observed physical and physiological conditions in primary care, respectively, and they co-occur in 30–50% [9]. Notably, pain-related sensory pathways have been shown to share similar structures of the brain engaged in mood control [10–14], and depressive states may facilitate the modulation of pain in some structures [15–17]. These data suggest that there are neuroplastic maladaptive changes that would lead to the onset of chronic pain-induced depression. At this point of comorbidity between chronic pain and depression, pharmacological treatment of the patient becomes difficult. For this reason, it is crucial to study the neural mechanisms underlying this comorbidity. In addition to depressive disorders, patients suffering from chronic pain have been shown to exert increased anxiety and fear, and the presence of both might enhance their suffering from pain [18]. Anxiety and chronic pain conditions share underlying cognitive and behavioral mechanisms, such as exacerbated attention to threat and anxious avoidance of physical effort [19, 20]. It is hypothesized that pain catastrophizing and fear of movement present in people with OA lead to activity avoidance [21]. Studies have shown that more than half of examined patients with OA who underwent total knee arthroplasty (TKA) reported being afraid of falling [22], and increased helplessness has been associated with increased knee pain severity in people with knee OA [23]. As the basic research of OA relies mostly on in vivo models, we used the MIA injection to the knee to induce the OA symptoms in rats. The MIA-based model is well-established and mirrors both the histological and pain-related characteristics of human degenerative OA [24]. Therefore, to better understand the comorbity and the underlying mechanisms of OA and affective symptoms, we performed the longitudinal study of the presence of depressive and anxiety symptoms after MIA injections in rats. Behavioral tests were supplemented by neurochemical assessment of monoamines in the frontal cortex (FCX), striatum (STR), and nucleus accumbens (NAS) since these structures have been shown to modulate affective and motivational aspects of pain and increasing levels of monoamines is the most popular treatment strategy for depression and anxiety [25, 26]. Regarding the engagement of dopamine (DA) signaling in both chronic pain and affective disorders [27], we assessed DA and its metabolites levels in abovementioned brain structures. To shed more light on the neurocircuitry affected by chronic pain, we employed computational methods, such as graph analysis and feature selection algorithms, which allowed us to establish key neuromodulatory drivers in the circuit.

## Materials and methods

### Animals

**Fig. 1:**
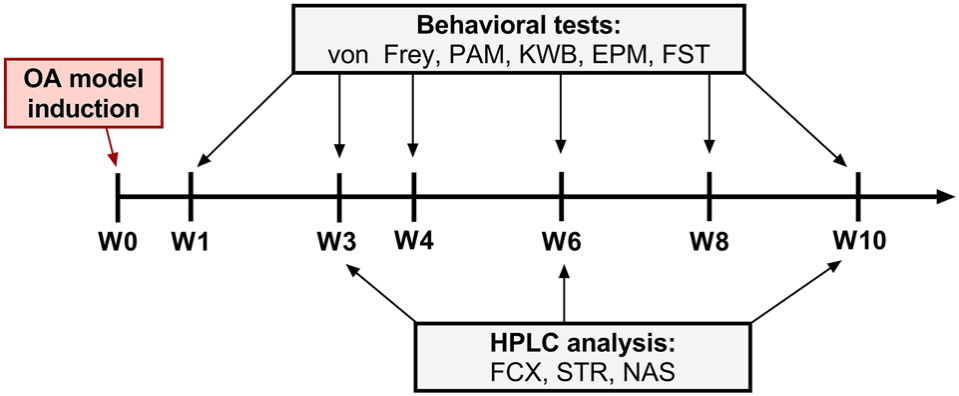
Representative timeline of conducted experiments

Male Wistar (Charles River, Sulzfeld, Germany) 7-week-old rats (initially weighing 225–250 g) were used in this study. Male rats were selected for the experiment to minimize variability related to the estrous cycle, as it was presented to affect anxiety-related behavior [28]. All animal experiments were conducted in accordance with the National Research Council’s Guide for the Care and Use of Laboratory Animals and were approved by the Local Bioethics Committee of the Institute of Pharmacology (Kraków, Poland, approval number 279/2021). Separate cohorts of animals were used for behavioral (*N* = 16) and biochemical (*N* = 48) studies. Animals were housed four per cage under a 12/12-h light/dark cycle with food and water available ad libitum and enriched environment. All experiments were performed between 9:00 and 12:00. Sample size was estimated using power analysis, resulting in experimental groups consisting of *N* = 8, assuming significance cut-off = 0.05, large effect size = 1, and power = 0.75. The behavioral tests were performed as follows: von Frey test, pressure application measurement, kinetic weight-bearing (on day 1 of the testing week, at an interval within 1 h), open field (on day 2), elevated plus maze (on day 3), forced swimming test (at day 5) (Fig. 1). All experiments were performed by experimenters blinded to the treatment conditions. No animal was excluded or died during the time of experiments.

### OA model induction

On day 0, rats were briefly anesthetized with 5% isoflurane (Forane®, Baxter Healthcare Corporation, Deerfield, IL, USA) in the oxygen (3 L/min). Joint damage was induced by a single intra-articular (*ia*) injection of sodium monoiodoacetate (MIA; 2 mg/50 µL; Sigma-Aldrich, St. Louis, MO, USA) dissolved in 0.9% saline into the rear right knee using a Hamilton syringe (250 µL, 1725 LT SYR) and a 30-G ½″ needle. Sham-operated animals received 50 µL of 0.9% saline *ia*. All surgical procedures were performed under sterile conditions. Animals were monitored for suspected adverse effects such as swelling or unexpected adverse effects but none were observed.

### Von Frey test

At 1, 2, 4, 6, 8, and 10 weeks post-OA model induction, three behavioral tests were performed to assess the pain threshold of rats. Calibrated Von Frey monofilaments (Bioseb, Vitrolles, France) were used to measure tactile allodynia. Rats were placed in Plexiglas cages with a wire net floor 5 min before the experiment. Filaments were applied to the mid-plantar surface of the ipsilateral hind paw according to the ascending method [29]. Each filament was applied five times for an approximately 2- to 3-s period. The monofilament that evoked at least two responses was noted as the paw withdrawal threshold. The strength of the von Frey monofilament bending forces was as follows: 0.6, 1.0, 1.4, 2, 4, 6, 8, 10, 15, and 26 g as a cut-off for response.

### Pressure application measurement (PAM) test

A pressure application measurement (PAM) device (PAM; Ugo Basile, Comerio Varese, Italy) has been used for the assessment of joint hyperalgesia [30]. The animals were held lightly, and the operator placed a thumb with a force transducer mounted unit on one side of the animal’s knee joint. A gradually increasing squeeze force was applied across the joint at a rate of approximately 30 g/s with a maximum test duration of 15 s or 500 g of applied force. The test was stopped when the animal showed signs of pain and distress, such as limb withdrawal, wriggling or vocalizing, and the peak gram force (gf) was applied immediately before limb withdrawal was noted.

### Kinetic weight-bearing (KWB) test

To characterize differences in weight-bearing during walking, a kinetic weight-bearing (KWB) instrument (Bioseb, Vitrolles, France) was used. Sensors placed on the ground measured the weight borne by each individual paw during the walking sequence of a freely moving animal, while a built-in camera detected the center of gravity of the animal. Data collection was terminated when five validated runs were obtained or after 6 min of acquisition. If the animal did not run during this time window, the measurement was repeated at the end of the session. Rats that failed to make at least one validated run during the second session were excluded from the analysis. The presented results describe the difference in peak force (the mean of the maximum forces of each rear paw measured in centinewtons, cN) between the rear right and rear left paws.

### Elevated plus maze (EPM) test

The plus-shaped apparatus (Ugo Basile, Comerio Varese, Italy), elevated to a height of 60 cm, consisted of two opposite open arms (50 × 10 cm) and two opposite closed arms (50 × 10 × 40 cm), extending from the central platform (10 × 10 cm). The animal was placed at the center of the apparatus facing the open arm and was allowed to freely explore the plus maze for 5 min. The test was conducted under low-intensity light (20 lx). The number of open arm entries, number of closed arm entries, the percentage of time spent in open arms, and locomotor activity (measured as a traveled distance) were recorded by a camera placed above the maze and scored automatically using Any-maze® tracking software. The apparatus was cleaned with 70% ethanol and dried after each trial.

### Forced swim test (FST)

FST performance was based on the method of Porsolt [31] and performed as described in the paper by Antkiewicz-Michaluk et al. [32]. Briefly, the test consisted of a pretest session (on day 1) and the main test (on day 2). On the first day, rats were individually placed for 15 min in a nontransparent, plastic cylinder (Ø 23 cm, height 50 cm) filled with water (25–26 °C) up to 30 cm in height. Twenty-four hours later (on the main-test session day), the procedure was repeated, and the animals were placed in water for 5 min to assess the time of behaviors: immobility (floating on the surface), climbing (energetic movements of limbs with forepaws breaking the walls of the cylinder), and active swimming. The water in the cylinder was changed after each testing trial. The duration times of the aforementioned behaviors were manually scored by experimenters blinded to the experimental conditions.

### Open field (OF) test

The OF test was performed using a black, wooden box (60 × 60 × 25 cm) divided into 16 equal squares (15 × 15 cm). The four inner squares constituted the inner zone (30 × 30 cm), and the remaining squares (each stretching 15 cm against the walls of the apparatus) formed the outer zone. The animals were placed in the center of the box and allowed to freely explore the apparatus for 10 min under dimly lit (20 lx) conditions. The entries into the inner zone, time spent in the inner zone, and locomotor activity (measured as a traveled distance) were recorded by a camera above the apparatus and automatically scored by Any-maze® tracking software. The apparatus was cleaned with 70% ethanol and dried after each testing trial.

### HPLC analysis

In a particular time point (3, 6 or 10 week), animals were killed by decapitation. FCX, STR, and NAS were dissected and frozen on solid CO_2_ (− 70 °C) and stored until biochemical assays. DA along with its metabolites 3,4-dihydroxyphenylacetic acid (DOPAC), 3-methoxytyramine (3-MT), and homovanillic acid (HVA, the final metabolite); 5-HT along with its metabolite 5-hydroxyindoleacetic acid (5-HIAA); and NA along with its metabolite normetanephrine (NM) were assayed by means of high-performance liquid chromatography (HPLC) with electrochemical detection (Antec DECADE Elite, Alphen a/d Rijn, The Netherlands). The chromatograph (Shimadzu, SCL-40, Kyoto, Japan) was equipped with C18 column. The sample preparation procedure was based on our previous protocol [33].

### Statistical analysis

The statistical analysis was performed using Prism software, version 6 (GraphPad Software). Data were first examined for Gaussian distribution by the Shapiro–Wilk normality test and the equality of variances by the Brown–Forsythe test. Normally distributed data with equal variances were analyzed using two-way ANOVA with Šidák’s post hoc test for comparison of the treatment effects. The number of animals used in specific experiments is denoted under the graphs. No exclusion critera were pre-determined and all animals were included in the final analysis. Outlier values greater/less than the mean ± 2 * standard deviations were excluded from the analysis. The data were considered significant only when *p* < 0.05*.* Detailed description of statistical analysis for each experiment is provided in the Supplementary material.

### Correlation and network analysis

A correlation matrix between neurotransmitter levels in different structures was created in R software (R version 4.1.1) with Pearson’s test from the Hmisc package (version 4.7.2). For the analysis, we have used all measured neurotransmitter levels in all the researched structures and the data were pooled across all timepoints (3, 6, and 10 weeks). Visualization of the correlation matrix was performed with the corrplot package (version 0.92), whereas only significant correlations with *p* < 0.05 were visualized and used for downstream network analysis. False discovery rate was used to correct for multiple comparisons and minimize type 1 error. A network graph was created with the igraph package (version 1.3.5) based on correlation values, |*r*| > 0.3 and visualized in Cytoscape (version 3.9.1). We employed the Boruta algorithm (version 8.0.0) and vertex degree to identify influential nodes in the network [34]. The Boruta algorithm is a wrapper algorithm around a random forest that allows for the extraction of independent variables that significantly explain the dependent variable; in this case, MIA versus sham treatment. We analyzed correlations and constructed graphs separately for neurotransmitters and their metabolites to avoid obvious, strong correlations between them. We then constructed graphs of correlation in both treatment groups separately (sham and MIA). Network analysis was conducted in Cytoscape software, version 3.9.1. The average number of neighbors indicates the average connectivity of a node in the network. A normalized version of this parameter is the network density, which is defined as the proportion of edges in a network relative to the total number of possible edges ($D=\frac{2\left|E\right|}{\left|V\right|\left(\left|V\right|-1\right)}$).

## Results

### Development of pain phenotype during OA development

**Fig. 2:**
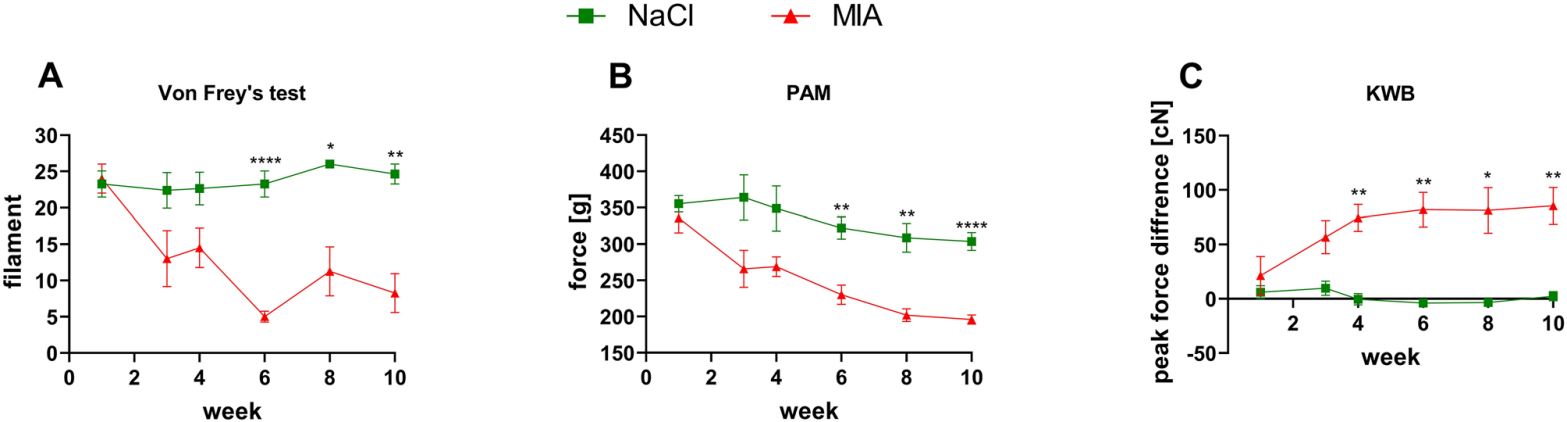
Tactile allodynia (**A**), joint hyperalgesia (**B**), and difference in weight-bearing (**C**) in OA rats (MIA) or sham-operated controls (NaCl). At week 0, rats were injected (*ia*) with NaCl (0.9% saline) or MIA (2 mg). Paw sensitivity was measured by Von Frey’s, PAM, and KWB tests 1, 3, 4, 6, 8, and 10 weeks post-MIA injection. Two-way ANOVA (or mixed model) and Šidák’s post hoc test were performed, and groups contained *N* = 8 animals. Data are presented as mean ± SEM. * denotes *p* < 0.05; ** denotes *p* < 0.01; *** denotes *p* < 0.001; **** denotes *p* < 0.0001 difference between NaCl and MIA rats. *ia*, intra-articular; KWB, kinetic weight-bearing; PAM, pressure application measurement; MIA, monoiodoacetate; OA, osteoarhritis

Sham-operated animals did not show any signs of allodynia throughout the whole experimental period, while a significant, gradual increase in mechanical sensitivity (therefore a decrease in withdrawal threshold to touch stimulus) was observed in MIA-treated rats (effect of time: *F*_3.11,43.54_ = 4.1, *p* = 0.0112; effect of treatment: *F*_1,14_ = 43.47, *p* < 0.0001; interaction of time and treatment: *F*_5,70_ = 5.04, *p* = 0.0005; post hoc analysis:; *p* < 0.0001 in week 6; *p* = 0.0196 in week 8; *p* = 0.0015 in week 10) (Fig. 2A, Suppl. Tab. 1). A significant difference between sham and MIA animals was also observed in joint hyperalgesia measurements in the PAM test (Fig. 2B). OA animals had significantly lower pain thresholds than control rats from week 6 until week 10 (effect of time: *F*_3.53,49.39_ = 7.96, *p* < 0.0001; effect of treatment: *F*_1,14_ = 51.74, *p* < 0.0001; interaction of time and treatment: *F*_5,70_ = 1.56, *p* = 0.1837; post hoc analysis: *p* = 0.003 in week 6; *p* = 0.0039 in week 8; *p* < 0.0001 in week 10) (Fig. 2B, Suppl. Tab. 1). Similarly, in the KWB test, control rats equally weighed both rear paws until 10 weeks of the experiment (Fig. 2C). A significant difference between the tested groups was observed from week 4 until the end of the experiment (effect of time: *F*_3.54,57.39_ = 1.48, *p* = 0.2245; effect of treatment: *F*_1,81_ = 84.46, *p* < 0.0001; interaction of time and treatment: *F*_5,81_ = 2.64, *p* = 0.0292; post hoc analysis: *p* = 0.0018 in week 4; *p* = 0.0051 in week 6; *p* = 0.0377 in week 8; *p* = 0.0086 in week 10) (Fig. 2C, Suppl. Tab. 1).

### Pain induces depressive- and anxiety-like behavior

**Fig. 3:**
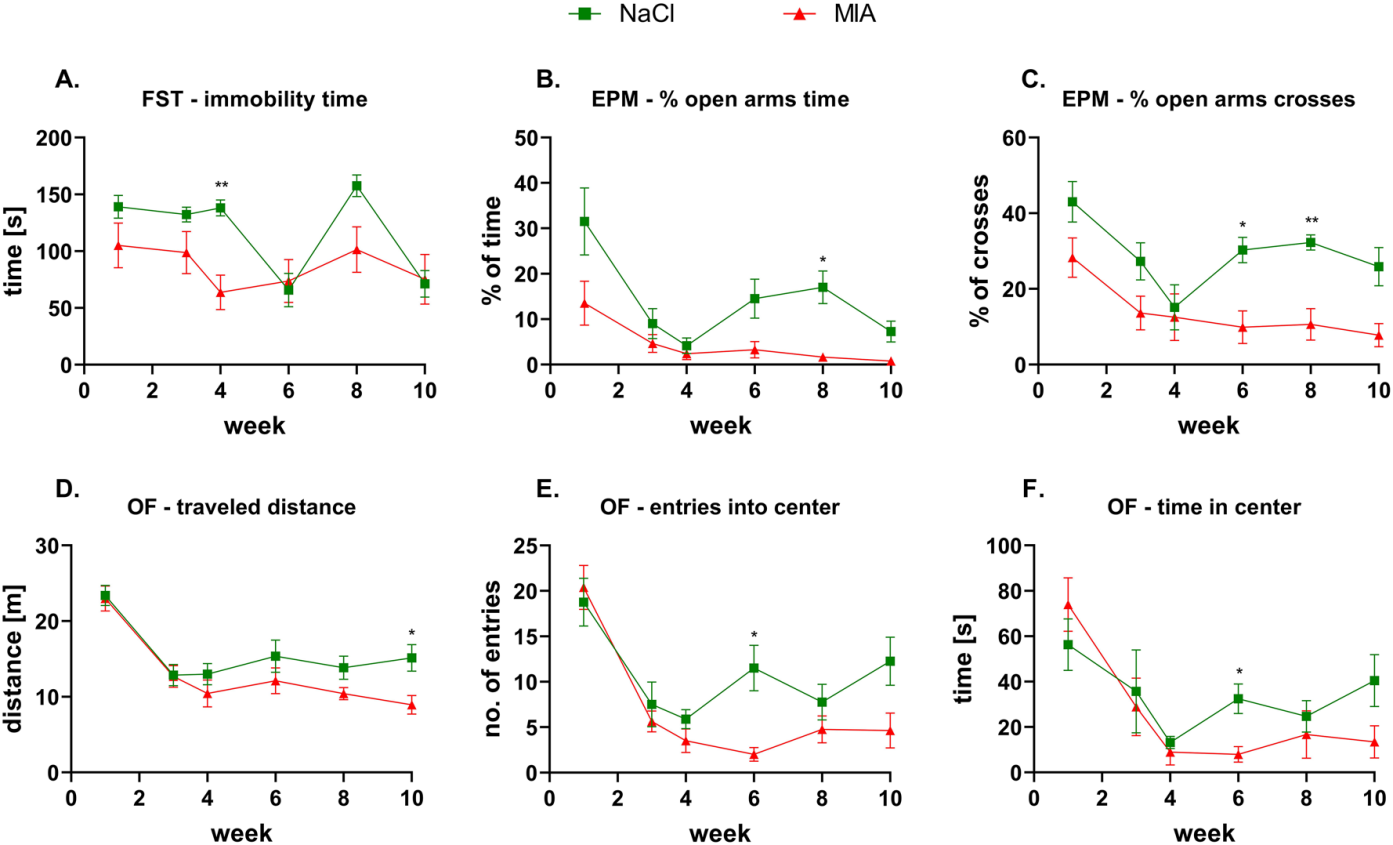
Depressive and anxiety behavior analysis. At week 0, rats were injected (*ia*) with NaCl (0.9% saline) or MIA (2 mg). Time of immobility in the forced swim test (**A**); % of time spent in the open arms (**B**) and % of open crosses (**C**) in the elevated plus maze; traveled distance in the center zone (**D**), number of entries into the center zone (**E**), and time spent in the center zone (**F**) in the open field were measured 1, 3, 4, 6, 8, and 10 weeks after OA model induction. Two-way ANOVA (or mixed model) and Šidák’s post hoc analysis were performed, and groups contained *N* = 8 animals. Data are presented as mean ± SEM. * denotes *p* < 0.05; ** denotes *p* < 0.01 between the NaCl and MIA groups. *ia*, intra-articular; EPM, elevated plus maze; FST, forced swim test; MIA, monoiodoacetate; OA, osteoarthritis; OF, open field

In the FST, we found significantly decreased (effect of time: *F*_3.11,43.55_ = 14.69, *p* < 0.0001; effect of treatment: *F*_1,14_ = 2.94, *p* = 0.1085; interaction of both factors: *F*_5,70_ = 6.04, *p* = 0.0001; post hoc analysis: *p* = 0.0074) immobility time in MIA-treated rats at week 4 compared to the control group. Other time points showed no differences in depressive-like behavior measured by time of immobility (Fig. 3A, Suppl. Tab. 1). In the EPM test, a significantly smaller percentage of time spent in the open arms was observed at week 8 (*p* = 0.0202) in MIA-treated animals than in the control group (effect of time: *F*_2.1,29.42_ = 12.05, *p* = 0.0001; effect of treatment: *F*_1,14_ = 9.48, *p* = 0.0082; interaction of time and treatment: *F*_5,70_ = 2.52, *p* = 0.0372) (Fig. 3B, Suppl. Tab. 1). In addition, a significant decrease in the percentage of open arm crosses (effect of time: *F*_3.1,43.38_ = 6.82, *p* = 0.0006; effect of treatment: *F*_1,14_ = 14.79, *p* = 0.0018; interaction of time and treatment: *F*_5,70_ = 1.43, *p* = 0.2252) was observed at weeks 6 and 8 (post hoc analysis: *p* = 0.0149 and *p* = 0.0049, respectively) in the MIA group (Fig. 3C, Suppl. Tab. 1). No significant changes were observed at other experimental time points (Fig. 3B, C). We did not find significant differences between sham and MIA groups in the total (open + closed) number of arms entries (Suppl. Fig. 1, Suppl. Tab. 1).

OF test analysis showed that MIA-treated animals exhibited significantly decreased traveled distance at week 10 (effect of time: *F*_5,70_ = 31.22, *p* < 0.0001; effect of treatment: *F*_1,14_ = 2.62, *p* = 0.1278; interaction of both factors: *F*_5,70_ = 1.98, *p* = 0.0929; post hoc analysis: *p* = 0.0341) (Fig. 3D, Suppl. Tab. 1). At week 6, the MIA group showed a decreased number of entries into the center zone (effect of time: *F*_2.48,34.15_ = 24.4, *p* < 0.0001; effect of treatment: *F*_1,14_ = 3.53, *p* = 0.0814; interaction of time and treatment: *F*_5,69_ = 3.09, *p* = 0.0142; post hoc analysis: *p* = 0.0376) (Fig. 3E, Suppl. Tab. 1) and decreased time spent in the center area (effect of time: *F*_2.6,35.88_ = 12.13, *p* < 0.0001; effect of treatment: *F*_1,14_ = 0.55, *p* = 0.4725; interaction of both factors: *F*_5,69_ = 1.98, *p* = 0.092; post hoc analysis: *p* = 0.0413) (Fig. 3F, Suppl. Tab. 1). We did not observe other substantial changes in the analyzed parameters at any other time point (Suppl. Tab. 1).

### Persistent OA pain induces monoamines and their metabolite changes in the rat brain

#### The levels of monoamines in the right FCX

**Fig. 4:**
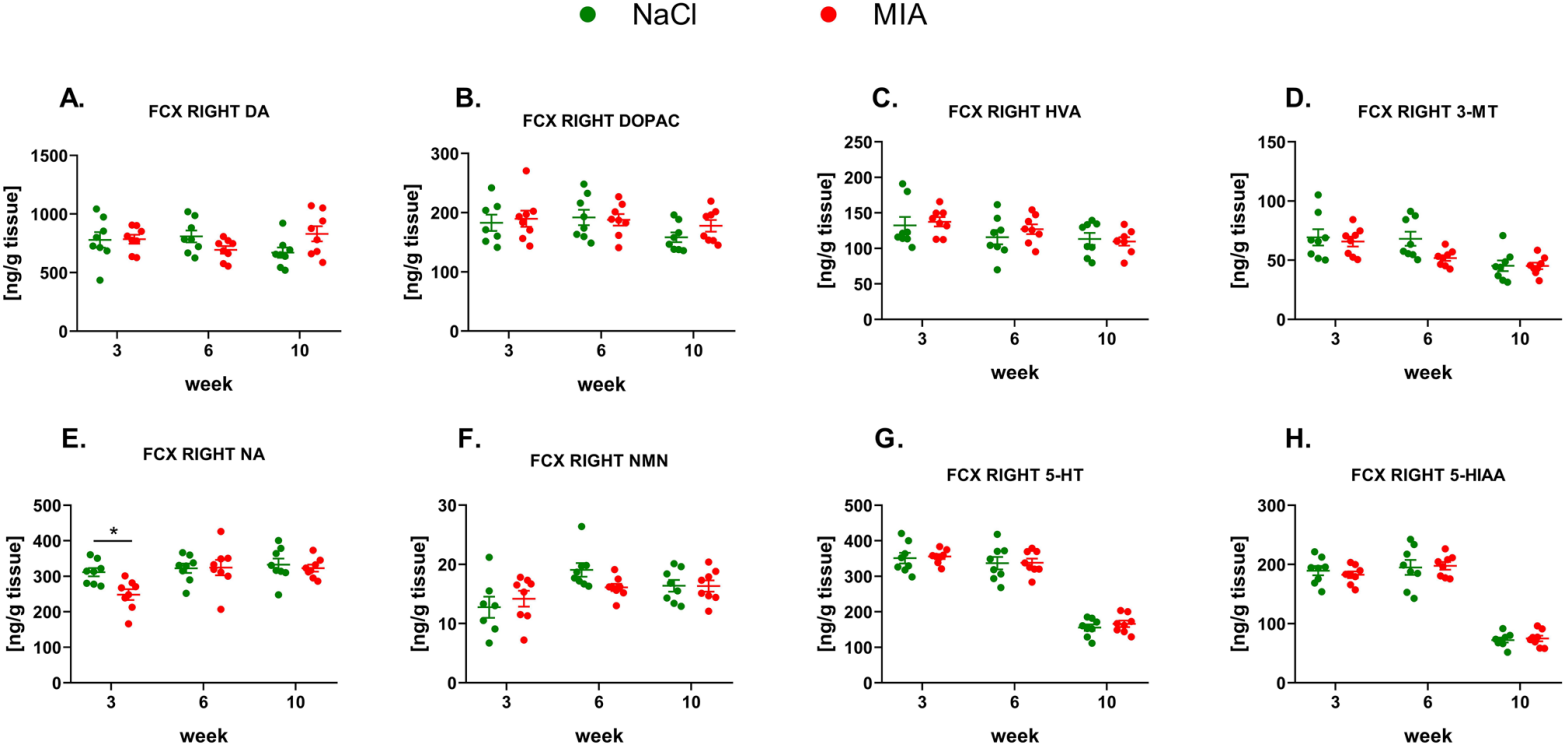
Monoamines DA (**A**), NA (**E**), and 5-HT (**G**) and their metabolite levels (**B**, **C**, **D**, **F**, **H**) measured in the right FCX by HPLC. At week 0, rats were injected (*ia*) with NaCl (0.9% saline) or MIA (2 mg). Rats were decapitated at weeks 3, 6, 10 and right FCX was dissected for further HPLC analysis. Two-way ANOVA (or mixed model) and Šidák’s post hoc analysis were performed, and groups contained *N* = 8 animals. Data are presented as mean ± SEM, separate dots represent value in individual rats. * denotes *p* < 0.05 between the NaCl and MIA groups. 3-MT, 3-methoxytyramine; 5-HIAA, 5-hydroxyindoleacetic acid; 5-HT, serotonin; *ia*, intra-articular; DA, dopamine; DOPAC, 3,4-dihydroxyphenylacetic acid; FCX, frontal cortex; HVA, homovanillic acid; HPLC, high-performance liquid chromatography; MIA, monoiodoacetate; NA, noradrenaline; NAS, nucleus accumbens; NM, normetanephrine; OA, osteoarthritis; STR, striatum

Statistical analysis showed insignificant changes between treatment groups in the levels of DA and its metabolites in the right FCX (Fig. 4A–D, Suppl. Tab. 2). However, we observed a significant decrease (effect of time: *F*_1.78,24.96_ = 6.0, *p* = 0.0092; effect of treatment: *F*_1,14_ = 3.68, *p* = 0.0757; interaction of both factors: *F*_2,28_ = 2.56, *p* = 0.0954; post hoc analysis: *p* < 0.05) in NA levels in MIA-treated animals at week 3, with no changes in NM levels at any time point (Fig. 4E, F, Suppl. Tab. 2). In addition, 5-HT and 5-HIAA levels did not change significantly in MIA animals compared to the control group (Fig. 4G, H, Suppl. Tab. 2).

#### The levels of monoamines in the left FCX

**Fig. 5:**
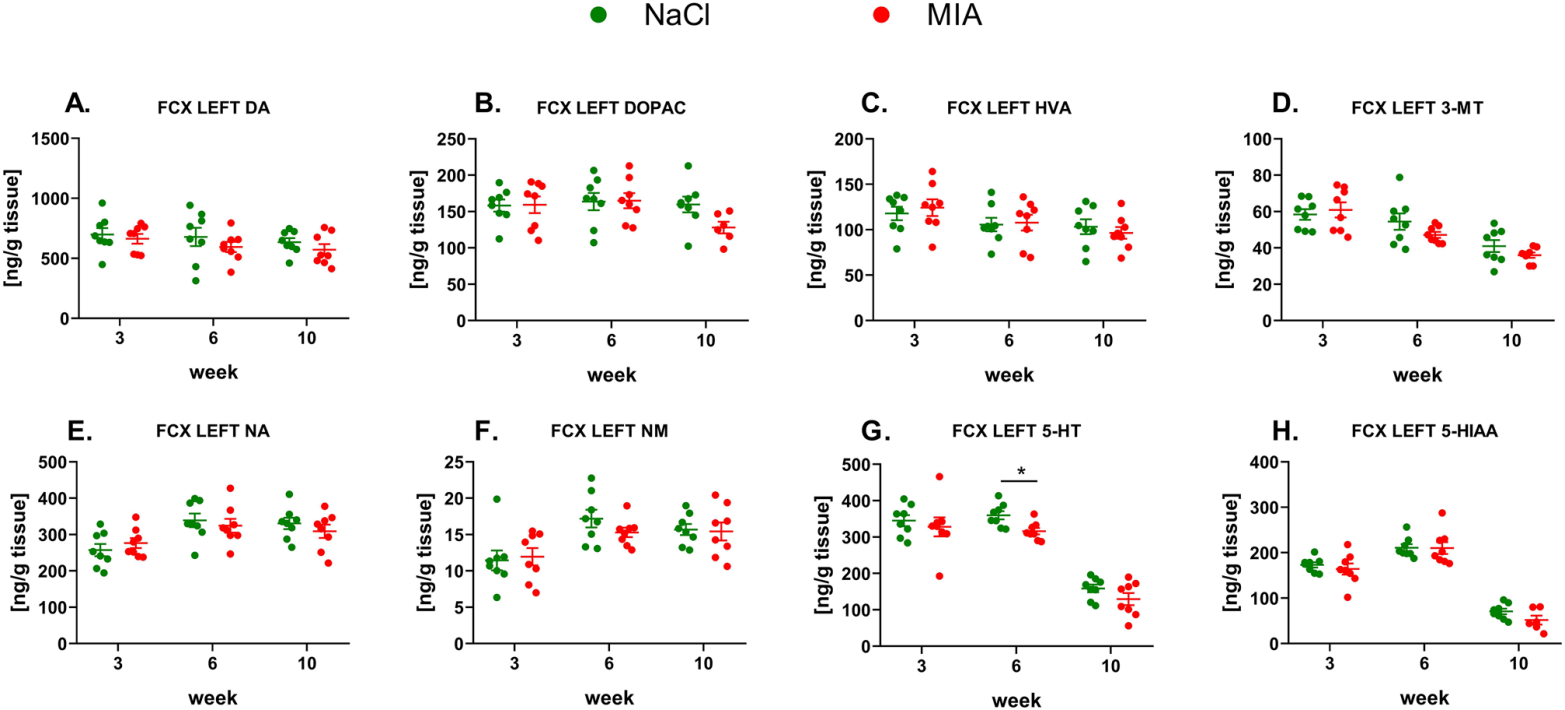
Monoamines DA (**A**), NA (**E**), and 5-HT (**G**) and their metabolite levels (**B**, **C**, **D**, **F**, **H**) measured in the left FCX by HPLC. At week 0, rats were injected (*ia*) with NaCl (0.9% saline) or MIA (2 mg). Rats were decapitated at weeks 3, 6, 10 and left FCX was dissected for further HPLC analysis. Two-way ANOVA (or mixed model) and Šidák’s post hoc analysis were performed, and groups contained *N* = 8 animals. Data are presented as mean ± SEM, separate dots represent value in individual rats. * denotes *p* < 0.05 between the NaCl and MIA groups. 3-MT, 3-methoxytyramine; 5-HIAA, 5-hydroxyindoleacetic acid; 5-HT, serotonin; *ia*, intra-articular; DA, dopamine; DOPAC, 3,4-dihydroxyphenylacetic acid; FCX, frontal cortex; HVA, homovanillic acid; HPLC, high-performance liquid chromatography; MIA, monoiodoacetate; NA, noradrenaline; NAS, nucleus accumbens; NM, normetanephrine; OA, osteoarthritis; STR, striatum

We did not observe significant changes in DA, NA or their metabolite levels in the left FCX (Fig. 5A–F, Suppl. Tab. 3). A significant decrease (effect of time: *F*_1.7,23.84_ = 90.12, *p* < 0.0001; effect of treatment: *F*_1,14_ = 6.72, *p* = 0.0213; interaction of time and treatment: *F*_2,28_ = 0.33, *p* = 0.7246), post hoc analysis: *p* < 0.05) in the 5-HT concentration in MIA-treated animals was observed at week 6 of the experiment in the left FCX (Fig. 5G, Suppl. Tab. 3); however, the levels of its metabolite, 5-HIAA, did not differ significantly between the MIA and control groups at any time point (Fig. 5H, Suppl. Tab. 3).

#### The levels of monoamines in the right STR

**Fig. 6:**
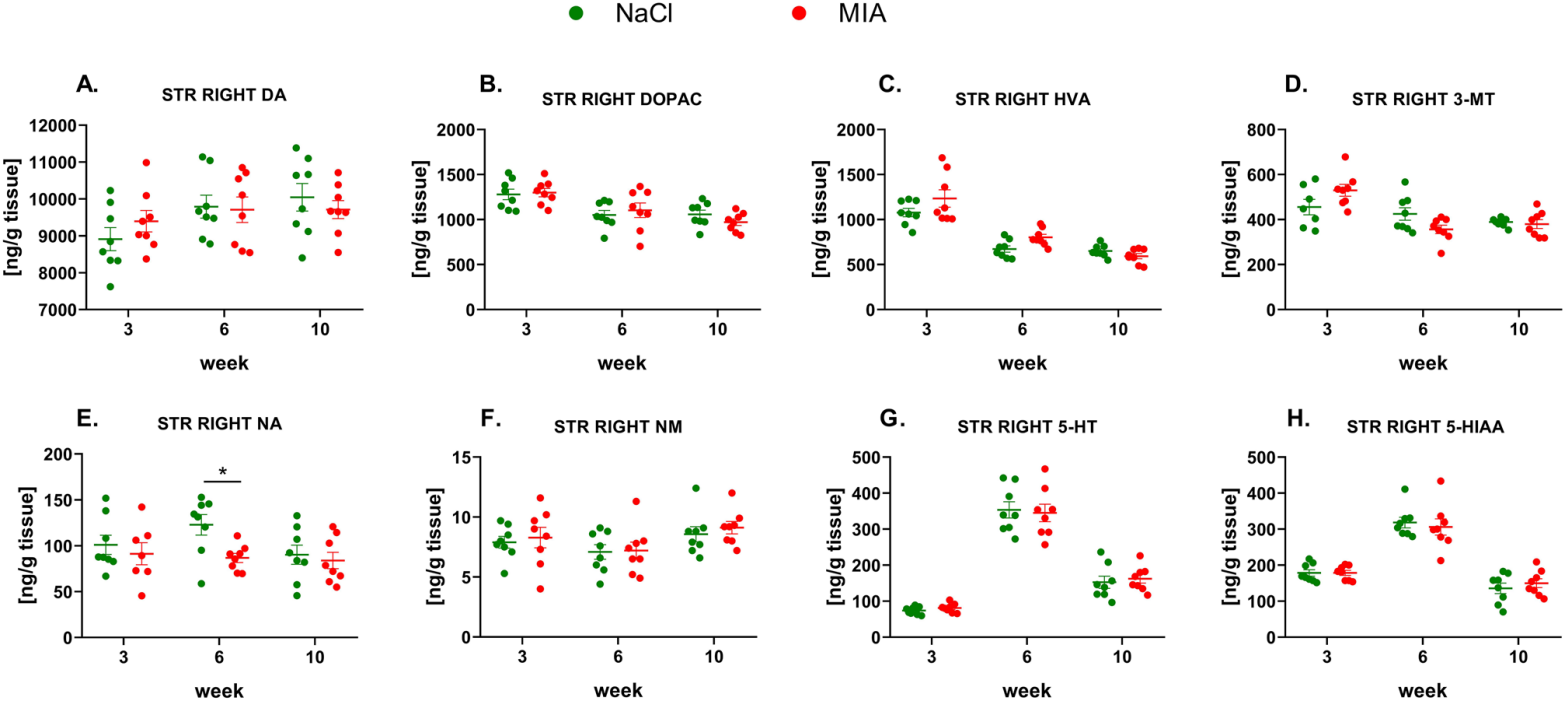
Monoamines DA (**A**), NA (**E**), and 5-HT (**G**) and their metabolite levels (**B**, **C**, **D**, **F**, **H**) measured in the right STR by HPLC. At week 0, rats were injected (*ia*) with NaCl (0.9% saline) or MIA (2 mg). Rats were decapitated at weeks 3, 6, 10 and right STR was dissected for further HPLC analysis. Two-way ANOVA (or mixed model) and Šidák’s post hoc analysis were performed, and groups contained *N* = 8 animals. Data are presented as mean ± SEM, separate dots represent value in individual rats. * denotes *p* < 0.05 between the NaCl and MIA groups. 3-MT, 3-methoxytyramine; 5-HIAA, 5-hydroxyindoleacetic acid; 5-HT, serotonin; *ia*, intra-articular; DA, dopamine; DOPAC, 3,4-dihydroxyphenylacetic acid; FCX, frontal cortex; HVA, homovanillic acid; HPLC, high-performance liquid chromatography; MIA, monoiodoacetate; NA, noradrenaline; NAS, nucleus accumbens; NM, normetanephrine; OA, osteoarthritis; STR, striatum

Statistical analysis showed no significant changes in DA or its metabolite concentrations at any of the tested time points (Fig. 6A–D, Suppl. Tab. 4). We observed a significant decrease in the NA level in the MIA group at week 6 of the experiment (effect of time: *F*_1.99,26.82_ = 2.05, *p* = 0.1488; effect of treatment: *F*_1,14_ = 3.42, *p* = 0.0855; interaction of both factors: *F*_2,27_ = 1.72, *p* = 0.199, post hoc analysis: *p* < 0.05) (Fig. 6E, Suppl. Tab. 4); however, the amount of NM did not differ between the control and MIA-treated animals (Fig. 6F, Suppl. Tab. 4). The levels of 5-HT and 5-HIAA did not differ among the tested groups at any of the experimental time points. (Fig. 6G, H, Suppl. Tab. 4).

#### The levels of monoamines in the left STR

**Fig. 7:**
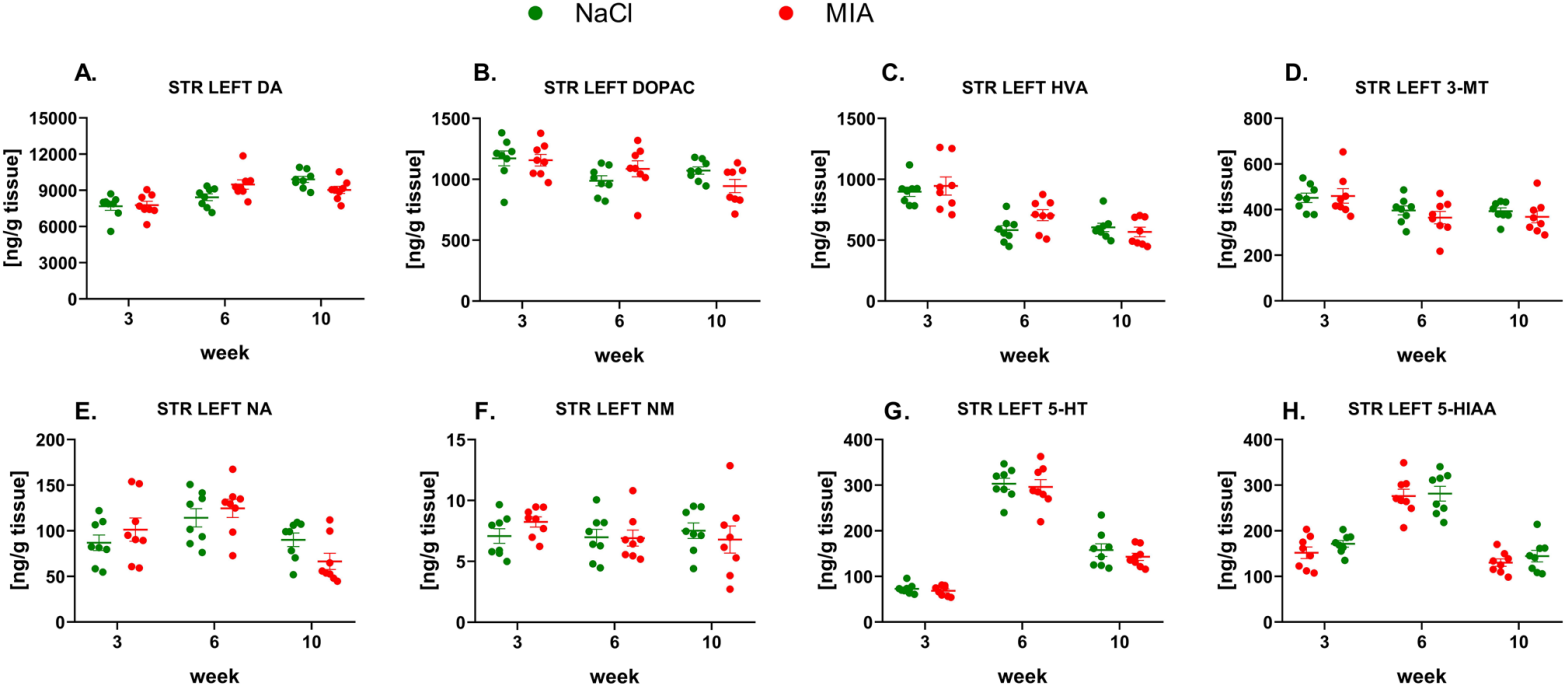
Monoamines DA (**A**), NA (**E**), and 5-HT (**G**) and their metabolite levels (**B**, **C**, **D**, **F**, **H**) measured in the left STR by the HPLC. At week 0, rats were injected (*ia*) with NaCl (0.9% saline) or MIA (2 mg). Rats were decapitated at weeks 3, 6, 10 and left STR was dissected for further HPLC analysis. Two-way ANOVA (or mixed model) and Šidák’s post hoc analysis were performed, and groups contained *N* = 8 animals. Data are presented as mean ± SEM, separate dots represent value in individual rats. 3-MT, 3-methoxytyramine; 5-HIAA, 5-hydroxyindoleacetic acid; 5-HT, serotonin; *ia*, intra-articular; DA, dopamine; DOPAC, 3,4-dihydroxyphenylacetic acid; FCX, frontal cortex; HVA, homovanillic acid; HPLC, high-performance liquid chromatography; MIA, monoiodoacetate; NA, noradrenaline; NAS, nucleus accumbens; NM, normetanephrine; OA, osteoarthritis; STR, striatum

We observed no substantial changes in the levels of any of the tested monoamines or their metabolites at any time points in the left STR (Fig. 7A–H, Suppl. Tab. 5).

#### The levels of monoamines in the right NAS

**Fig. 8:**
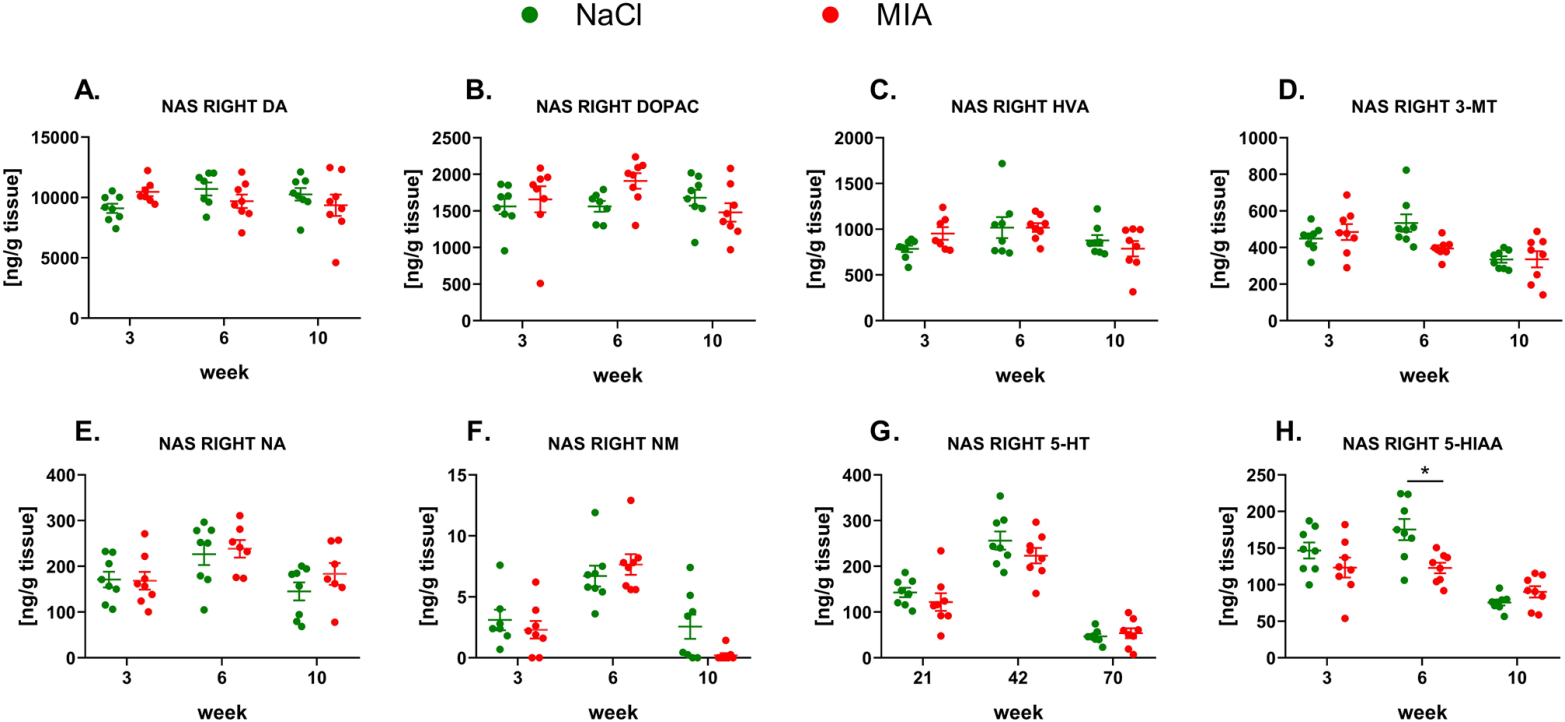
Monoamines DA (**A**), NA (**E**), and 5-HT (**G**) and their metabolite levels (**B**, **C**, **D**, **F**, **H**) measured in the right NAS by HPLC. At week 0, rats were injected (*ia*) with NaCl (0.9% saline) or MIA (2 mg). Rats were decapitated at weeks 3, 6, 10 and right NAS was dissected for further HPLC analysis. Two-way ANOVA (or mixed model) and Šidák’s post hoc analysis were performed, and groups contained *N* = 8 animals. Data are presented as mean ± SEM, separate dots represent value in individual rats. * denotes *p* < 0.05 between the NaCl and MIA groups. 3-MT, 3-methoxytyramine; 5-HIAA, 5-hydroxyindoleacetic acid; 5-HT, serotonin; *ia*, intra-articular; DA, dopamine; DOPAC, 3,4-dihydroxyphenylacetic acid; FCX, frontal cortex; HVA, homovanillic acid; HPLC, high-performance liquid chromatography; MIA, monoiodoacetate; NA, noradrenaline; NAS, nucleus accumbens; NM, normetanephrine; OA, osteoarthritis; STR, striatum

In the right NAS, DA and NA and their metabolite levels did not differ significantly among the tested groups at any time point (Fig. 8A–F. Suppl. Tab. 6). However, we observed a significant decrease (effect of time: *F*_1.99,27.83_ = 26.06, *p* < 0.0001; effect of treatment: *F*_1,14_ = 4.72, *p* = 0.0476; interaction of both factors: *F*_2,28_ = 6.06, *p* = 0.0065; post hoc analysis: *p* < 0.05) in the 5-HIAA level in the MIA-treated rats at week 6 (Fig. 8H, Suppl. Tab. 6), while the concentration of 5-HT did not differ significantly among the tested groups at the experimental time points (Fig. 8G, Suppl. Tab. 6).

#### The levels of monoamines in the left NAS

**Fig. 9:**
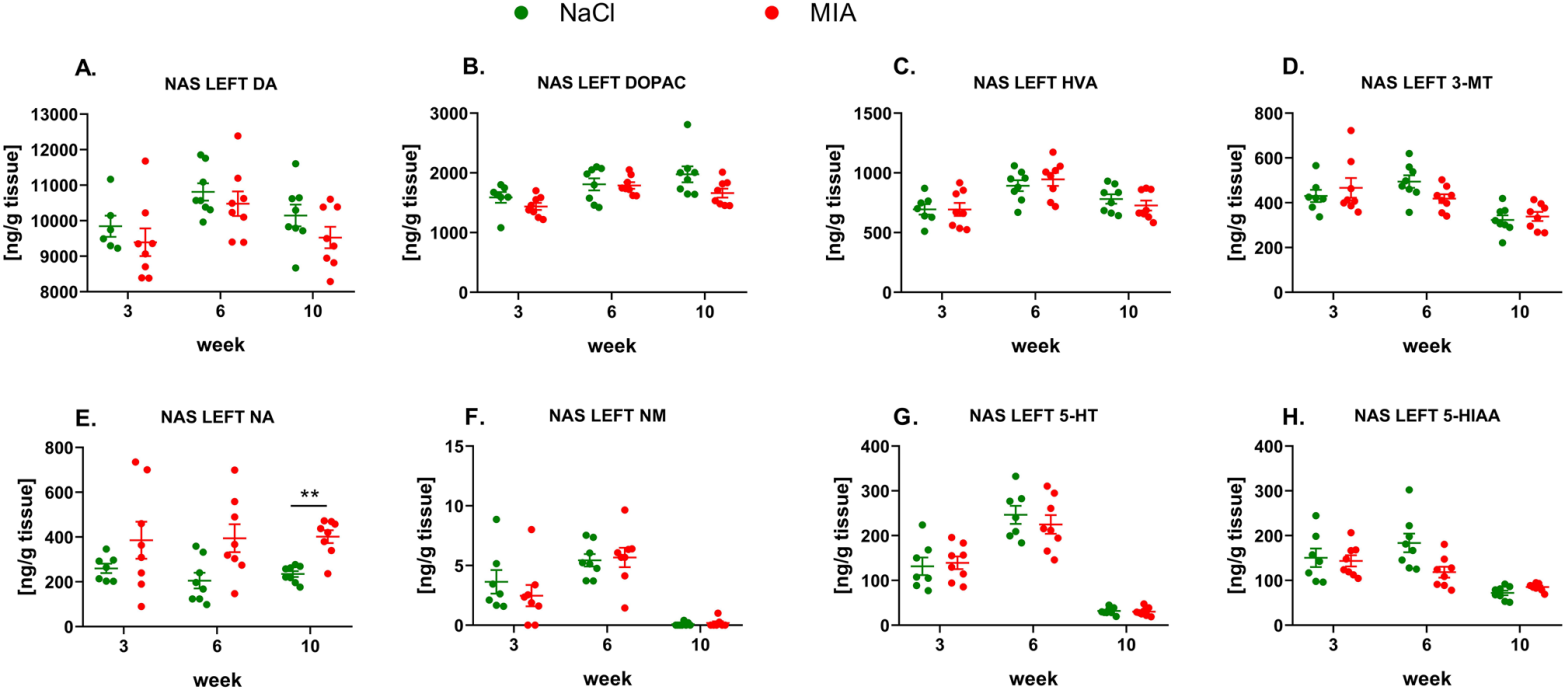
Monoamines DA (**A**), NA (**E**), and 5-HT (**G**) and their metabolite levels (**B**, **C**, **D**, **F**, **H**) measured in the left NAS by HPLC. At week 0, rats were injected (*ia*) with NaCl (0.9% saline) or MIA (2 mg). Rats were decapitated at weeks 3, 6, 10 and left NAS was dissected for further HPLC analysis. Two-way ANOVA (or mixed model) and Šidák’s post hoc analysis were performed, and groups contained *N* = 8 animals. Data are presented as mean ± SEM, separate dots represent value in individual rats. ** denotes *p* < 0.01 between the NaCl and MIA groups. 3-MT, 3-methoxytyramine; 5-HIAA, 5-hydroxyindoleacetic acid; 5-HT, serotonin; *ia*, intra-articular; DA, dopamine; DOPAC, 3,4-dihydroxyphenylacetic acid; FCX, frontal cortex; HVA, homovanillic acid; HPLC, high-performance liquid chromatography; MIA, monoiodoacetate; NA, noradrenaline; NAS, nucleus accumbens; NM, normetanephrine; OA, osteoarthritis; STR, striatum

Statistical analysis showed no significant changes in DA or its metabolite levels in the left NAS (Fig. 9A–D, Suppl. Tab. 7). However, we observed a substantial increase in NA concentrations in MIA-treated rats at week 10 (effect of time: *F*_1.53,31.31_ = 0.13, *p* = 0.8268; effect of treatment: *F*_1,14_ = 16.74, *p* = 0.0002; interaction of both factors: *F*_2,41_ = 0.22, *p* = 0.8037; post hoc test: *p* < 0.01) (Fig. 9E, Suppl. Tab. 7), whereas NM levels remained unchanged among the tested groups (Fig. 9F, Suppl. Tab. 7). The changes in levels of 5-HT and 5-HIAA were shown to be insignificant at all experimental time points (Fig. 9G, H, Suppl. Tab. 7).

### Visualization of the correlation matrix for neurotransmitters and metabolites during the development of OA

**Fig. 10:**
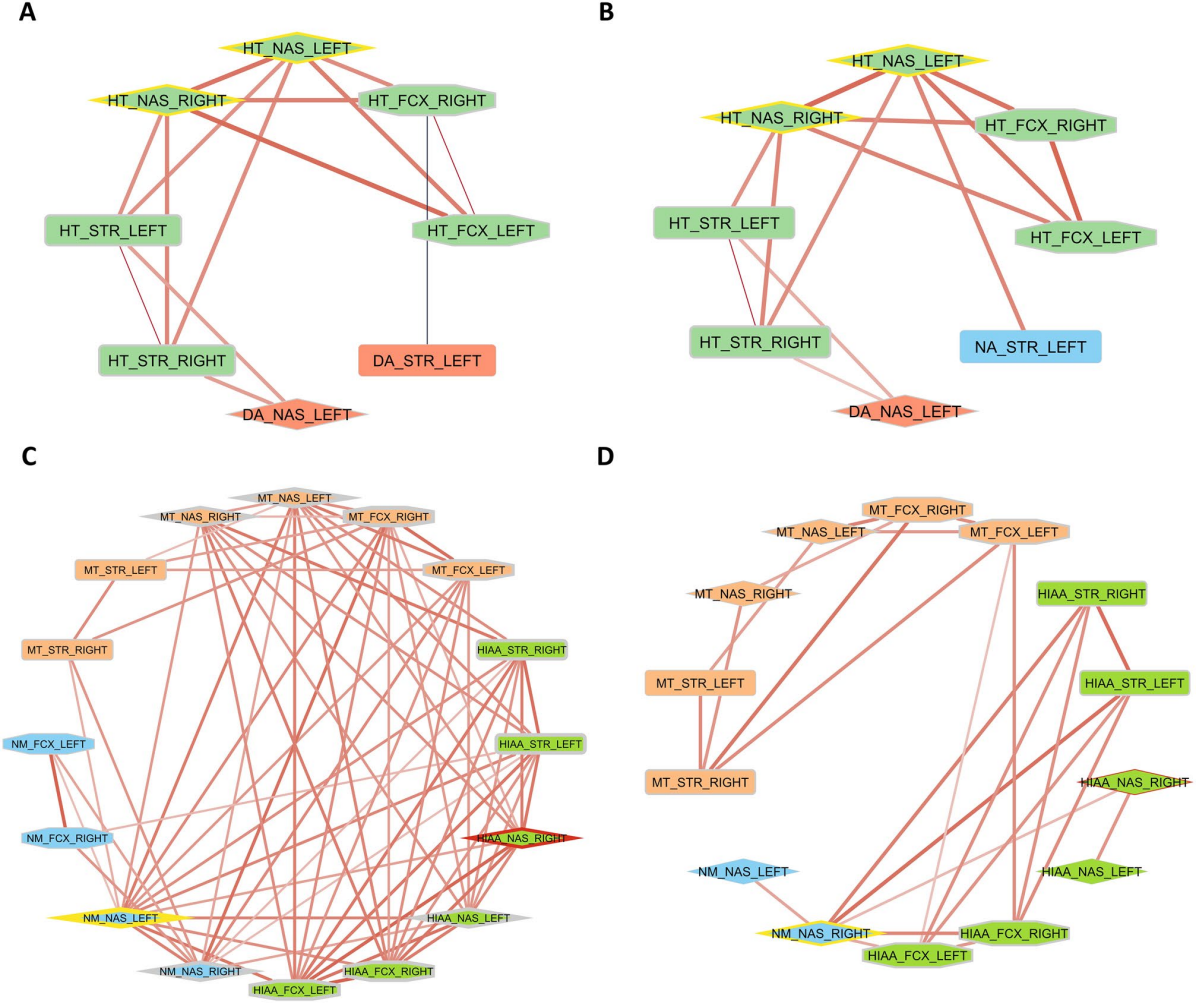
Correlation networks based on Pearson’s analysis in different treatment groups with *N* = 8 across all timepoints (3, 6 and 10 weeks). The graph presents correlations with |*r*| > 0.3 and *p* < 0.05 in sham (**A**, **C**) and MIA (**B**, **D**) animals. Red denotes a positive correlation, whereas blue denotes a negative correlation. Edge width represents the strength of the correlation. Yellow borders denote nodes with the highest vertex degree, while red borders denote nodes selected by the Boruta algorithm. 3-MT, 3-methoxytyramine; 5-HIAA, 5-hydroxyindoleacetic acid; 5-HT, serotonin; DA, dopamine; FCX, frontal cortex; MIA, monoiodoacetate; NA, noradrenaline; NAS, nucleus accumbens; NM, normetanephrine; STR, striatum

The Boruta algorithm identified NA levels in the left NAS and 5-HIAA levels in the right NAS as significant predictors of the treatment (MIA or sham). Based on correlation analysis, we constructed a network of interactions of either neurotransmitters or metabolites in different structures (Fig. 10). For neurotransmitters, there was no difference in graph parameters between MIA and sham animals (Fig. 10A, B). In both the MIA and sham graphs, the 5-HT level in NAS was the most interconnected node (Fig. 10A, B). In the sham correlation network of metabolites, the density value was equal to 0.558 and contained 16 nodes with 67 edges (*r* > 0.3 and *p* < 0.05) and an average number of neighbors equal to 8.375 (Fig. 10C). In contrast, in the MIA network of metabolite correlations, the density value was equal to 0.264. It contained 14 nodes with only 24 edges (*r* > 0.3 and *p* < 0.05) and an average number of neighbors equal to 3.429 (Fig. 10 D). In the sham group, the highest degree was found for the NA metabolite NM in the left NAS (13 incident edges), which was highly correlated with both DA and 5-HT metabolites (yellow borders, Fig. 10C). A distinctive feature of MIA treatment, 5-HIAA levels in the right NAS, showed nine incident edges in the sham network (red border, Fig. 10C). In the MIA network, the node represented by NM in the right NAS was the most interconnected node with the highest degree (yellow borders, Fig. 10D). A distinctive feature of MIA treatment, 5-HIAA levels in the right NAS, was only positively correlated with 5-HIAA levels in the left NAS and the most interconnected node, NM, in the right NAS (red border, Fig. 10D). Bilaterally, 3-MT and 5-HIAA in the NAS and FCX were highly correlated with each other in sham animals (Fig. 10C) but not in MIA animals (Fig. 10D). In addition, NM levels in the NAS were highly correlated with 3-MT and 5-HIAA in all three structures (NAS, FCX, and STR) only in sham animals (Fig. 10C) but not in MIA animals (Fig. 10D).

## Discussion

In this study, for the first time, we investigated the development of pain, depressive, and anxiety behaviors within 10 weeks after OA model induction as comorbid symptoms in relation to changes in the brain of monoamines and their metabolite levels in rats.

Over the course of 10 weeks, we observed the development of pain phenotype, as revealed by the Von Frey, PAM, and KWB tests. Interestingly, pain development was accompanied by anxiety-like behavior in the OF and EPM tests, although depressive-like behavior was absent in the FST. It is important to mention that FST, similar to other tests, was performed on the same group of rats as we employed this paradigm to reduce the number of animals involved in the study. However, it may be recognized as a possible confounder, as exposure to FST itself is interpereted as a stress [35]. In the study of Mezadri et al. [36], it has been shown that repeated exposure to FST can change the coping strategy of animals facing the stressor. We observed significantly lowered immobility time in week 4 in MIA animals, what in the context of the study may indicate disturbed coping mechanisms in OA animals at this point. In the context of anxiety behaviors, we observed in both groups the tendency to decline the measured parameters of EPM and OF between week 1 and 4 (Fig. 3B, C, E, F). It may be related to re-exposure of animals to the tests and this phenomenon has been described in details by [37]. Interesingly, the anxiety-like behavior occurred in 6 and 8 weeks post-MIA injection (Fig. 3B, C, E, F) and this fact may be explained by a slight, however important, decrease of frontal 5-HT level at week 6 in MIA animals (Fig. 5G). For example, Sang et al. [38] showed that increased activity of 5-HT transporter in prefrontal cortex may be responsible for development of anxiety behaviors in neuropathic rats, whereas injection of 5-HT to this region suppressed this phenomenon.

In addition, performing HPLC analysis of key monoamine levels and their metabolites in three structures related to the limbic system—the FCX, STR and NAS—we revealed a decrease in 5-HT neurotransmission in the right NAS. Moreover, there was a decrease in NA neurotransmission in the right FCX and STR but an increase in the left NAS. Surprisingly, we failed to observe a strict pattern of changes in the monoamine neurotransmission over the 10-week experiment that might shed more light to the development of anxiety and depression accompying chronic pain. Significant changes were observed unilaterally at single time points, suggesting more complicated mechanism of comorbidity of pain and affective disorders. Therefore, explaining behavioral changes by the engagement of particular neurotransmitters pathways over 10 weeks of persisting pain may be insufficient. Despite this fact, the Boruta algorithm distinguished changes in the NAS as significant predictors of MIA treatment, suggesting that those changes were more consistent throughout the time course of OA development. Presented results indicating NAS as an important structure for pain processing are in line with previous research which have shown that NAS is both receiving and sending nociceptive signals. Furthermore, NAS shares connections with variety of brain regions involved in cognition and emotion, controlling affective behavior [39].

Our previous research has already examined a link between monoamine levels and pain development in the MIA model [40]. To shed more light on the neurochemical changes underlying affective symptoms of OA, we performed graph and correlation analyses since they have shown that chronic pain can affect brain connectivity [41]. First, we revealed that MIA treatment disrupts the otherwise highly synchronized network of monoamine metabolites. Moreover, graph analysis further indicated the significance of NAS neurotransmission as the key node, highly intercorrelated with neurotransmitters in other structures. In MIA animals, a decrease in 5-HIAA levels but not 5-HT, as measured by the index [5-HIAA]/[5-HT], suggests that there was a decrease in the synaptic release of 5-HT in the NAS, which in turn might affect monoaminergic neurotransmission in the rest of the brain. For example, serotonergic inputs reduce excitatory postsynaptic currents of medium spiny neurons in the NAS [42].

In contrast, higher levels of NA in the left NAS were another significant feature of MIA treatment. Moreover, basal levels of NA metabolite in the NAS were highly intercorrelated with both 5-HT and DA in all three structures in healthy animals but not in MIA animals. These results suggest that both 5-HT and NA neurotransmission in the NAS are mostly affected by MIA treatment and might be responsible for anxiety-like behavior. In humans, functional magnetic resonance imaging (fMRI) activity in the NAS appears to reflect the affective and motivational aspects of pain. A study performed by Vania Apkarian’s group revealed that NAS activation in response to painful stimuli is potentiated in patients with chronic back pain [43]. Moreover, these authors also revealed that chronic pain increases connectivity between NAS and FCX [43] and decreases gray matter volume in the NAS [44]. In addition, spared nerve injury increases the excitability of NAS spiny projection neurons and alters their synaptic connectivity [45]. A recent study by Manz et al. [46] showed the involvement of NA neurotransmission in NAS microcircuitry. We also detected a significant decrease in NA concentrations in the right STR in MIA animals, in line with a study by Taylor et al. [47], who showed that, although NA content in the ventral striatum was significantly increased in neuropathic animals, higher levels of NA were correlated with lower levels of pain. Further pharmacological studies with specific modulation of 5-HT and NA levels in the NAS or STR should be able to reveal the relevance and functional implications of these findings.

Interestingly, one study explains the findings of our research quite well. In 2001, Espejo and Miñano [48] showed that DA depletion in the FCX increases NA neurotransmission in the NAS, also accompanied by anxiety-like behavior and hyperlocomotion that could possibly explain the decrease in immobility time in the FST in our study. In fact, drugs that increase NA levels, such as desipramine, reduce the immobility time in the FST [49]. The literature regarding DA content in the FCX during chronic pain has been conflicting, reporting both decreases and no changes [50, 51]. However, at the functional level, tail-pinch stressors also elicit DA release in the PFC [52]. Moreover, it has been shown that DA release in the PFC increases the activity of mPFC neurons projecting to the ventrolateral periaqueductal gray (vlPAG) matter and reduces mechanical hypersensitivity during neuropathic pain states [53]. Conversely, the NAS receives glutamatergic inputs from FCX [47], while deep brain stimulation of the NAS core can lead to increases in DA, NA, and 5-HT in FCX [54].

In summary, our study revealed anxiety-like behavior in an animal model of OA, adding to our knowledge about the face validity of the MIA model. Moreover, network analysis and feature selection algorithms allowed us to identify 5-HT and NA neurotransmission in the NAS as the main node affected by chronic pain development. Providing neurochemical underpinnings to previous fMRI results from clinical findings, our data pave the way toward understanding the functional roles of NA and 5-HT in the NAS in chronic pain. Future studies in this area could bring novel treatment strategies to aid chronic pain patients.

### Supplementary Information

Below is the link to the electronic supplementary material.

Supplementary file1 (DOCX 825 KB)

## Data Availability

The datasets generated during and/or analyzed during the current study are not publicly available but are available from the corresponding author on reasonable request.

## Abbreviations

3-MT: 3-Methoxytyramine
5-HIAA: 5-Hydroxyindoleacetic acid
5-HT: Serotonin
DA: Dopamine
DOPAC: 3,4-Dihydroxyphenylacetic acid
EPM: Elevated plus maze
FCX: Frontal cortex
fMRI: Functional magnetic resonance imaging
FST: Forced swim test
gf: Gram force
HPLC: High-performance liquid chromatography
HVA: Homovanillic acid
KWB: Kinetic weight-bearing
MIA: Monoiodoacetate
NA: Noradrenaline
NAS: Nucleus accumbens
NM: Normetanephrine
OA: Osteoarthritis
OF: Open field
PAM: Pressure application measurement
STR: Striatum
TKA: Total knee arthroplasty
vlPAG: Ventrolateral periaqueductal gray
