# Supplementary material for: Network analysis of monoamines involved in anxiety-like behavior in a rat model of osteoarthritis
Source: Pharmacol Rep. 2024 Jan 5;76(1):72–85. doi: 10.1007/s43440-023-00562-5 (PMC10830664; doi:10.1007/s43440-023-00562-5)
Supplement: Supplementary file 1 — Supplementary file1 (DOCX 825 KB) [file 43440_2023_562_MOESM1_ESM.docx]

**Supplementary data**

**Suppl. Figure 1.**





**Suppl. Fig. 1: Total number of entries to open and closed arms in Elevated Plus Maze Test in OA rats (MIA) or sham-operated controls (NaCl).** At week 0, rats were injected (*ia*) with NaCl (0.9% saline) or MIA (2 mg). Two-way ANOVA and Šidák’s post hoc test did not show significant differences between sham and MIA animals in total number of entries to arms of the apparatus measured 1, 3, 4, 6, 8 and 10 weeks post-MIA injection. Data are shown as mean ± SEM, N = 8 animals/group.

**Suppl. Table 1.**

| **BEHAVIORAL TESTS** | | | | | | | | | | |
| --- | --- | --- | --- | --- | --- | --- | --- | --- | --- | --- |
|  | **FST**  **Immobility time** | **EPM**  **% time in open arms** | **EPM**  **% open crosses** | **EPM**  **total arms entries** | **OF**  **distance in center zone** | **OF**  **number of entries into center zone** | **OF**  **time spent in center zone** | **Von Frey** | **PAM** | **KWB** |
| effect of *time* | F_3.11, 43.55_ = 14.69  *p<0.0001* | F_2.1, 29.42_ = 12.05  *p=0.0001* | F_3.1, 43.38_ = 6.82  *p=0.0006* | F_3.92_, _54.8_ = 5.19  *p* *= 0.0014* | F_5,70_ = 31.22  *p<0.0001* | F_2.48, 34.15_ = 24.4  *p<0.0001* | F_2.6, 35.88_ = 12.13  *p<0.0001* | F_3.11, 43.54_ = 4.1  *p=0.0112* | F_3.53, 49.39_ = 7.96  *p<0.0001* | F_3.54, 57.39_ = 1.48  *p=0.2245* |
| effect of *treatment* | F1_, 14_ = 2.94  *p=0.1085* | F_1, 14_ = 9.48  *p=0.0082* | F_1, 14_ = 14.79  *p=0.0018* | F_1,14_ = 0.35  *p = 0.5646* | F_1, 14_ = 2.62  *p=0.1278* | F_1, 14_ = 3.53  *p=0.0814* | F_1, 14_ = 0.55  *p=0.4725* | F_1, 14_ = 43.47  *p<0.0001* | F_1, 14_ = 51.74  *p=0<0.0001* | F_1, 81_ = 84.46  *p<0.0001* |
| interaction  *time x treatment* | F_5, 70_ = 6.04  *p=0.0001* | F_5, 70_ = 2.52  *p=0.0372* | F_5, 70_ = 1.43  *p=0.2252* | F_5,70_ = 1.09  *p = 0.3741* | F_5, 70_ = 1.98  *p=0.0929* | F_5, 69_ = 3.09  *p=0.0142* | F_5, 69_ = 1.98  *p=0.092* | F_5, 70_ = 5.04  *p=0.0005* | F_5, 70_ = 1.56  *p=0.1837* | F_5, 81_ = 2.64  *p=0.0292* |
| **Post hoc *p* value**  Week 1  Week 3  Week 4  Week 6  Week 8  Week 10 | *p = 0.6332*  *p = 0.5486*  *p = 0.0074*  *p = 0.9997*  *p = 0.1675*  *p > 0.9999* | *p = 0.3302*  *p = 0.8576*  *p = 0.9682*  *p = 0.2102*  *p = 0.0202*  *p =0.1515* | *p = 0.3514*  *p = 0.3115*  *p = 0.9998*  *p = 0.0149*  *p = 0.0049*  *p = 0.0595* | *p = 0.9223*  *p = 0.9883*  *p = 0.446*  *p > 0.9999*  *p = 0.9999*  *p = 0.9999* | *p > 0.9999*  *p > 0.9999*  *p = 0.8211*  *p = 0.6032*  *p = 0.5439*  *p = 0.0341* | *p = 0.9984*  *p = 0.9857*  *p = 0.6874*  *p = 0.0376*  *p = 0.8156*  *p = 0.2040* | *p = 0.8794*  *p = 0.9998*  *p = 0.987*  *p = 0.0413*  *p = 0.9902*  *p = 0.3492* | *p > 0.9999*  *p = 0.3275*  *p = 0.2113*  *p < 0.0001*  *p = 0.196*  *p = 0.0015* | *p = 0.963*  *p = 0.1607*  *p = 0.2201*  *p = 0.003*  *p = 0.0039*  *p < 0.0001* | *p = 0.9659*  *p = 0.1026*  *p = 0.0018*  *p = 0.0051*  *p = 0.0377*  *p = 0.0086* |

**Suppl. Tab. 1:** Detailed characteristic of statistical analysis (Two-way ANOVA or mixed analysis followed by Šidák’s post hoc test) of behavioral tests’ results (FST, EPM, OF, Von Frey, PAM, KWB).

**Suppl. Table 2.**

| **FCX RIGHT** | | | | | | | | |
| --- | --- | --- | --- | --- | --- | --- | --- | --- |
|  | **DA** | **DOPAC** | **3-MT** | **HVA** | **NA** | **NM** | **5-HT** | **5-HIAA** |
| effect of *time* | F_1.76, 24.64_ = 0.21  *p=0.7857* | F_1.96, 40.16_ = 2.10  *p=0.1372* | F_1.68, 23.46_ = 12.65  *p=0.0003* | F_1.65, 23.05_ = 5.08  *p=0.0196* | F_1.78, 24.96_ = 6.00  *p=0.0092* | F_1.77, 23.93_ = 7.07  *p=0.005* | F_1.68, 23.58_ = 126.30  *p<0.0001* | F_1.58, 22.13_ = 168.00  *p<0.0001* |
| effect of *treatment* | F_1, 14_ = 0.27  *p=0.6132* | F_1, 14_ = 0.62  *p=0.4353* | F_1, 14_ = 2.54  *p=0.1331* | F_1, 14_ = 0.24  *p=0.6286* | F_1, 14_ = 3.68  *p=0.0757* | F_1, 14_ = 0.25  *p=0.6239* | F_1, 14_ = 0.60  *p=0.4528* | F_1, 14_ = 0.01  *p=0.9331* |
| interaction  *time x treatment* | F_2,28_ = 3.13  *p=0.0594* | F_2, 41_ = 0.53  *p=0.5908* | F_2, 28_ = 1.79  *p=0.186* | F_2, 28_ = 0.50  *p=0.6104* | F_2, 28_ = 2.56  *p=0.0954* | F_2, 27_ = 2.04  *p=0.1501* | F_2, 28_ = 0.06  *p=0.9416* | F_2, 28_ = 0.28  *p=0.7585* |
| **Post hoc *p* value**  Week 3  Week 6  Week 10 | *p = 0.9998*  *p = 0.2268*  *p = 0.1732* | *p = 0.9809*  *p = 0.993*  *p = 0.3946* | *p = 0.9633*  *p = 0.0899*  *p > 0.9999* | *p = 0.9779*  *p = 0.7575*  *p = 0.9818* | *p = 0.0173*  *p = 0.9998*  *p = 0.9477* | *p = 0.8967*  *p = 0.1276*  *p > 0.9999* | *p = 0.9906*  *p > 0.9999*  *p = 0.8158* | *p = 0.864*  *p = 0.9972*  *p = 0.9637* |

**Suppl. Tab. 2**: Detailed characteristic of statistical analysis (Two-way ANOVA or mixed analysis followed by Šidák’s post hoc test) of monoamines (DA, NA, 5-HT) and their metabolites levels (DOPAC, 3-MT, HVA, NM and 5-HIAA) in right FCX.

**Suppl. Table 3.**

| **FCX LEFT** | | | | | | | | |
| --- | --- | --- | --- | --- | --- | --- | --- | --- |
|  | **DA** | **DOPAC** | **3-MT** | **HVA** | **NA** | **NM** | **5-HT** | **5-HIAA** |
| effect of *time* | F_1.68, 23.51_ = 1.44  *p=0.2563* | F_1.82, 23.72_ = 2.66  *p=0.0948* | F_1.49, 20.85_ = 33.33  *p<0.0001* | F_1.86, 25.98_ = 8.22  *p=0.0021* | F_1.56, 21.8_ = 7.68  *p=0.0051* | F_1.8, 25.22_ = 12.07  *p=0.0003* | F_1.7, 23.84_ = 90.12  *p<0.0001* | F_1.86, 24.12_ = 130.10  *p<0.0001* |
| effect of *treatment* | F_1, 14_ = 1.59  *p=0.2286* | F_1, 14_ = 0.94  *p=0.3485* | F_1, 14_ = 0.91  *p=0.36* | F_1, 14_ = 0.004  *p=0.9507* | F_1, 14_ = 0.18  *p=0.6772* | F_1, 14_ = 0.28  *p=0.6061* | F_1, 14_= 6.72  *p=0.0213* | F_1, 14_ = 1.34  *p=0.2662* |
| interaction  *time x treatment* | F_2, 28_ = 0.14  *p=0.8698* | F_2, 26_ = 2.12  *p=0.1402* | F_2, 28_ = 1.95  *p=0.16* | F_2, 28_ = 0.79  *p=0.4653* | F_2, 28_ = 0.77  *p=0.4717* | F_2, 28_ = 0.76  *p=0.4777* | F_2, 28_ = 0.33  *p=0.7246* | F_2, 26_ = 0.47  *p=0.6297* |
| **Post hoc *p* value**  Week 3  Week 6  Week 10 | *p = 0.9369*  *p = 0.7336*  *p = 0.6551* | *p = 0.9998*  *p = 0.9998*  *p = 0.1062* | *p = 0.9486*  *p = 0.4148*  *p = 0.4851* | *p = 0.934*  *p = 0.9971*  *p = 0.894* | *p = 0.7763*  *p = 0.9309*  *p = 0.7684* | *p = 0.9896*  *p = 0.4884*  *p = 0.9971* | *p = 0.9272*  *p = 0.0255*  *p = 0.4206* | *p = 0.882*  *p > 0.9999*  *p = 0.3544* |

**Suppl. Tab. 3:** Detailed characteristic of statistical analysis (Two-way ANOVA or mixed analysis followed by Šidák’s post hoc test) of monoamines (DA, NA, 5-HT) and their metabolites levels (DOPAC, 3-MT, HVA, NM and 5-HIAA) in left FCX.

**Suppl. Table 4.**

| **STR RIGHT** | | | | | | | | |
| --- | --- | --- | --- | --- | --- | --- | --- | --- |
|  | **DA** | **DOPAC** | **3-MT** | **HVA** | **NA** | **NM** | **5-HT** | **5-HIAA** |
| effect of *time* | F_1.85, 25.86_ = 3.91  *p=0.0358* | F_1.57, 22.03_ = 14.61  *p=0.0002* | F_1.71, 23.06_ = 13.73  *p=0.0002* | F_1.29, 18.01_ = 69.57  *p<0.0001* | F_1.99, 26.82_ = 2.05  *p=0.1488* | F_1.71, 23.93_ = 3.83  *p=0.0419* | F_1.17, 16.42_ = 125.50  *p<0.0001* | F_1.52, 21.3_ = 65.69  *p<0.0001* |
| effect of *treatment* | F_1, 14_ = 0.005  *p=0.9434* | F_1, 14_ = 0.01  *p=0.92* | F_1, 14_ = 0.004  *p=0.9519* | F_1, 14_ = 2.82  *p=0.1152* | F_1, 14_ = 3.42  *p=0.0855* | F1, 14 = 0.37  *p=0.5539* | F_1, 14_ = 0.07  *p=0.7932* | F_1, 14_ = 0.005  *p=0.9441* |
| interaction  *time x treatment* | F_2, 28_ = 1.14  *p=0.3355* | F_2, 28_= 0.93  *p=0.4079* | F_2, 27_ = 4.77  *p=0.016* | F_2, 28_ = 3.01  *p=0.0654* | F_2, 27_ = 1.72  *p=0.199* | F_2, 28_ = 0.07  *p=0.9372* | F_2, 28_ = 0.16  *p=0.8564* | F_2, 28_ = 0.37  *p=0.6971* |
| **Post hoc *p* value**  Week 3  Week 6  Week 10 | *p = 0.6231*  *p = 0.9972*  *p = 0.8528* | *p = 0.9923*  *p = 0.9306*  *p = 0.4287* | *p = 0.2969*  *p = 0.1772*  *p = 0.9628* | *p = 0.4393*  *p = 0.0508*  *p = 0.3798* | *p = 0.9095*  *p = 0.0438*  *p = 0.9588* | *p = 0.9737*  *p = 0.9992*  *p = 0.8822* | *p = 0.5426*  *p = 0.9923*  *p = 0.9571* | *p > 0.9999*  *p = 0.9589*  *p = 0.8507* |

**Suppl. Tab. 4:** Detailed characteristic of statistical analysis (Two-way ANOVA or mixed analysis followed by Šidák’s post hoc test) of monoamines (DA, NA, 5-HT) and their metabolites levels (DOPAC, 3-MT, HVA, NM and 5-HIAA) in right STR.

| **STR LEFT** | | | | | | | | |
| --- | --- | --- | --- | --- | --- | --- | --- | --- |
|  | **DA** | **DOPAC** | **3-MT** | **HVA** | **NA** | **NM** | **5-HT** | **5-HIAA** |
| effect of *time* | F_1.95, 27.3_ = 15.06  *p<0.0001* | F_1.68, 23.51_ = 5.72  *p=0.0126* | F_1.88, 26.34_ = 8.38  *p=0.0018* | F_1.77, 24.78_ = 32.77  *p<0.0001* | F_1.66, 23.18_ = 8.63  *p=0.0025* | F_1.68, 23.49_ = 0.63  *p=0.5147* | F_1.49, 20.91_ = 253.90  *p<0.0001* | F_1.92, 26.83_ = 70.64  *p<0.0001* |
| effect of *treatment* | F_1, 14_ = 0.14  *p=0.712* | F_1, 14_ = 0.11  *p=0.7496* | F_1, 14_ = 0.47  *p=0.5042* | F_1, 14_ = 1.10  *p=0.3113* | F_1, 14_ = 0.002  *p=0.9645* | F_1, 14_ = 0.03  *p=0.8562* | F_1, 14_ = 0.87  *p=0.3678* | F_1, 14_ = 1.82  *p=0.199* |
| interaction  *time x treatment* | F_2, 28_ = 4.36  *p=0.0224* | F_2, 28_ = 2.61  *p=0.0914* | F_2, 28_ = 0.51  *p=0.6058* | F_2, 28_ = 1.30  *p=0.2149* | F_2, 28_ = 2.19  *p=0.1304* | F_2, 28_ = 1.07  *p=0.3573* | F_2, 28_ = 0.13  *p=0.8745* | F_2, 28_ = 0.16  *p=0.8508* |
| **Post hoc *p* value**  Week 3  Week 6  Week 10 | *p = 0.9974*  *p = 0.1348*  *p = 0.1216* | *p = 0.9968*  *p = 0.5485*  *p = 0.1835* | *p = 0.9953*  *p = 0.7417*  *p = 0.8021* | *p = 0.9271*  *p = 0.1555*  *p = 0.867* | *p = 0.7455*  *p = 0.856*  *p = 0.1711* | *p = 0.3545*  *p = 0.9998*  *p = 0.9271* | *p = 0.8188*  *p = 0.9817*  *p = 0.7627* | *p = 0.5104*  *p = 0.9941*  *p = 0.7380* |

**Suppl. Table 5.**

**Suppl. Tab. 5:** Detailed characteristic of statistical analysis (Two-way ANOVA or mixed analysis followed by Šidák’s post hoc test) of monoamines (DA, NA, 5-HT) and their metabolites levels (DOPAC, 3-MT, HVA, NM and 5-HIAA) in left STR.

**Suppl. Table 6.**

| **NAS RIGHT** | | | | | | | | |
| --- | --- | --- | --- | --- | --- | --- | --- | --- |
|  | **DA** | **DOPAC** | **3-MT** | **HVA** | **NA** | **NM** | **5-HT** | **5-HIAA** |
| effect of *time* | F_1.79, 35.88_ = 0.32  *p=0.7033* | F_2, 27_ = 0.93  *p=0.4051* | F_1.63, 22.87_ = 10.65  p=0.001 | F_1.95, 26.29_ = 4.97  *p=0.0154* | F_1.95, 25.38_ = 8.55  *p=0.0015* | F_1.97, 26.62_ = 46.30  p<0.0001 | F_1.66, 23.17_ = 73.62  *p<0.0001* | F_1.99, 27.83_ = 26.06  *p<0.0001* |
| effect of *treatment* | F_1, 14_ = 0.15  *p=0.6969* | F_1, 14_ = 0.60  *p=0.4531* | F_1, 14_ = 1.15  *p=0.3025* | F_1, 14_ = 0.09  *p=0.7665* | F_1, 14_ = 0.71  *p=0.4124* | F_1, 14_ = 0.83  *p=0.3772* | F_1, 14_ = 2.30  *p=0.1520* | F_1, 14_ = 4.72  *p=0.0476* |
| interaction  *time x treatment* | F_2, 28_ = 2.66  *p=0.0823* | F_2, 27_ = 2.57  *p=0.0956* | F_2, 28_ = 4.02  *p=0.0293* | F_2, 27_= 1.87  *p=0.1734* | F_2, 26_ = 0.69  *p=0.5117* | F_2, 27_ = 3.44  *p=0.0467* | F_2, 28_ = 0.85  *p=0.4368* | F_2, 28_ = 6.06  *p=0.0065* |
| **Post hoc *p* value**  Week 3  Week 6  Week 10 | *p = 0.0603*  *p = 0.5127*  *p = 0.7818* | *p = 0.9573*  *p = 0.0579*  *p = 0.5726* | *p = 0.8658*  *p = 0.0671*  *p > 0.9999* | *p = 0.1629*  *p > 0.9999*  *p = 0.7781* | *p = 0.9995*  *p = 0.9737*  *p = 0.5579* | *p = 0.8619*  *p = 0.8251*  *p = 0.1451* | *p = 0.7338*  *p = 0.5388*  *p = 0.9252* | *p = 0.4937*  *p = 0.0252*  *p = 0.3146* |

**Suppl. Tab. 6:** Detailed characteristic of statistical analysis (Two-way ANOVA or mixed analysis followed by Šidák’s post hoc test) of monoamines (DA, NA, 5-HT) and their metabolites levels (DOPAC, 3-MT, HVA, NM and 5-HIAA) in right NAS.

**Suppl. Table 7.**

| **NAS LEFT** | | | | | | | | |
| --- | --- | --- | --- | --- | --- | --- | --- | --- |
|  | **DA** | **DOPAC** | **3-MT** | **HVA** | **NA** | **NM** | **5-HT** | **5-HIAA** |
| effect of *time* | F_1.84, 23.86_ = 5.98  *p=0.0091* | F_1.86, 38.16_ = 7.05  *p=0.003* | F_1.28, 26.13_ = 12.73  *p=0.0007* | F_1.61, 21.68_ = 12.24  *p=0.0006* | F_1.53, 31.31_ = 0.13  *p=0.8268* | F_1.72, 22.4_= 39.78  *p<0.0001* | F_1.31, 26.18_ = 92.26  *p<0.0001* | F_1.24, 25.33_ = 17.68  *p=0.0001* |
| effect of *treatment* | F_1, 14_ = 2.87  *p=0.1122* | F_1, 14_ = 4.88  *p=0.0328* | F_1, 14_ = 0.13  *p=0.7225* | F_1, 14_ = 0.0003  *p=0.9856* | F_1, 41_ = 16.74  *p=0.0002* | F_1, 14_ = 0.19  *p=0.6711* | F_1, 40_ = 0.17  *p=0.6818* | F_1, 41_ = 3.05  *p=0.088* |
| interaction  *time x treatment* | F_2, 26_ = 0.11  *p=0.894* | F_2, 41_ = 1.37  *p=0.2646* | F_2, 41_ = 2.28  *p=0.1155* | F_2, 27_ = 0.67  *p=0.5212* | F_2, 41_ = 0.22  *p=0.8037* | F_2, 26_ = 0.72  *p=0.4955* | F_2, 40_ = 0.48  *p=0.6222* | F_2, 41_ = 4.45  *p=0.0178* |
| **Post hoc *p* value**  Week 3  Week 6  Week 10 | *p = 0.7562*  *p = 0.8304*  *p = 0.4267* | *p = 0.4567*  *p = 0.9976*  *p = 0.1816* | *p = 0.8753*  *p = 0.1288*  *p = 0.9367* | *p > 0.9999*  *p = 0.8465*  *p = 0.7404* | *p = 0.4438*  *p = 0.0669*  *p = 0.0011* | *p = 0.7835*  *p = 0.993*  *p = 0.8845* | *p = 0.9845*  *p = 0.8527*  *p = 0.9725* | *p = 0.9897*  *p = 0.057*  *p = 0.1507* |

**Suppl. Tab. 7:** Detailed characteristic of statistical analysis (Two-way ANOVA or mixed analysis followed by Šidák’s post hoc test) of monoamines (DA, NA, 5-HT) and their metabolites levels (DOPAC, 3-MT, HVA, NM and 5-HIAA) in left NAS.
